# Offspring from Mouse Embryos Developed Using a Simple Incubator-Free Culture System with a Deoxidizing Agent

**DOI:** 10.1371/journal.pone.0047512

**Published:** 2012-10-09

**Authors:** Fumiaki Itoi, Mikiko Tokoro, Yukari Terashita, Kazuo Yamagata, Noritaka Fukunaga, Yoshimasa Asada, Teruhiko Wakayama

**Affiliations:** 1 Laboratory for Genomic Reprogramming, Center for Developmental Biology, RIKEN, Kobe, Japan; 2 The Asada Institute for Reproductive Medicine, Asada Ladies Clinic, Kasugai, Japan; 3 Laboratory of Animal Reproduction, Graduate School of Agricultural Science, Tohoku University, Sendai, Japan; 4 Center for Genetic Analysis of Biological Responses, Research Institute for Microbial Diseases, Osaka University, Suita, Japan; 5 Faculty of Life and Environmental Sciences, University of Yamanashi, Yamanashi, Japan, Kofu, Japan; Michigan State University, United States of America

## Abstract

To culture preimplantation embryos *in vitro*, water-jacketed CO_2_ incubators are used widely for maintaining an optimal culture environment in terms of gas phase, temperature and humidity. We investigated the possibility of mouse embryo culture in a plastic bag kept at 37°C. Zygotes derived from *in vitro* fertilization or collected from naturally mated B6D2F1 female mice were put in a drop of medium on a plastic culture dish and then placed in a commercially available plastic bag. When these were placed in an oven under air at 37°C for 96 h, the rate of blastocyst development and the cell numbers of embryos decreased. However, when the concentration of O_2_ was reduced to 5% using a deoxidizing agent and a small oxygen meter, most zygotes developed into blastocysts. These blastocysts were judged normal according to their cell number, Oct3/4 and Cdx2 gene expression levels, the apoptosis rate and the potential for full-term development after embryo transfer to pseudopregnant recipients. Furthermore, using this system, normal offspring were obtained simply by keeping the bag on a warming plate. This culture method was applied successfully to both hybrid and inbred strains. In addition, because the developing embryos could be observed through the transparent wall of the bag, it was possible to capture time-lapse images of live embryos until the blastocyst stage without needing an expensive microscope-based incubation chamber. These results suggest that mouse zygotes are more resilient to their environment than generally believed. This method might prove useful in economical culture systems or for the international shipment of embryos.

## Introduction

As mammalian embryos are very sensitive to the culture environment, it is important to maintain stable culture conditions [1]–[5]. Therefore, water-jacketed CO_2_ incubators are widely used for maintaining an optimal culture environment in terms of gas phase, temperature and humidity [6]. However, these incubators are based on earlier somatic tissue culture techniques [7] so they are large and heavy with an excess of culture space. Therefore, new concepts for incubators suitable for mammalian embryo culture have been developed in recent years. For example, benchtop or desktop incubators have been used successfully to culture human embryos [8]. However, these incubators are still large and require considerable installation space.

To address this problem, Vajta et al. developed a bovine preimplantation embryo culture system in which the culture dish was put into a foil bag and submerged in a water bath instead of being placed in a CO_2_ incubator (Submarine Incubation System: SIS) [9]. When bovine embryos cloned from somatic cell nuclei were cultured in this system, one healthy offspring was obtained [10]. However, Arias et al. reported that an evaluation of the quality of the blastocysts revealed that the number of cells was decreased, the rate of apoptosis increased and genes associated with the generation reactive oxygen species were upregulated in the bag culture system compared with the original incubator system [11]. Therefore, even though full-term offspring can be obtained from embryos cultured without a traditional CO_2_ incubator, it is not clear whether such embryos are damaged during the culture period.

Another obstacle to simplifying the embryo culture system is the gas phase. In general, it has been thought that mammalian embryos are sensitive to the gas phase used and that a continuous supply of CO_2_ is essential to maintain the good quality of the embryos. When mouse embryos were cultured in air (approximately 0.03% CO_2_), the pH of the medium was 8.1–8.3 and the embryos did not reach the blastocyst stage [12]. Ozawa et al. reported that a high pH in the medium caused a lower development rate and poorer embryo quality than traditional methods [13]. Therefore, chemical agents have been used to maintain the appropriate concentration of CO_2_ in small or portable incubators [14]–[18]. Using these conditions, bovine and murine embryos were cultured to the blastocyst stage with similar success rates as for the original CO_2_ incubator systems [19]. Thus, adjustment of the CO_2_ concentration using chemical agents is one solution for simplifying embryo culture.

**Figure 1:**
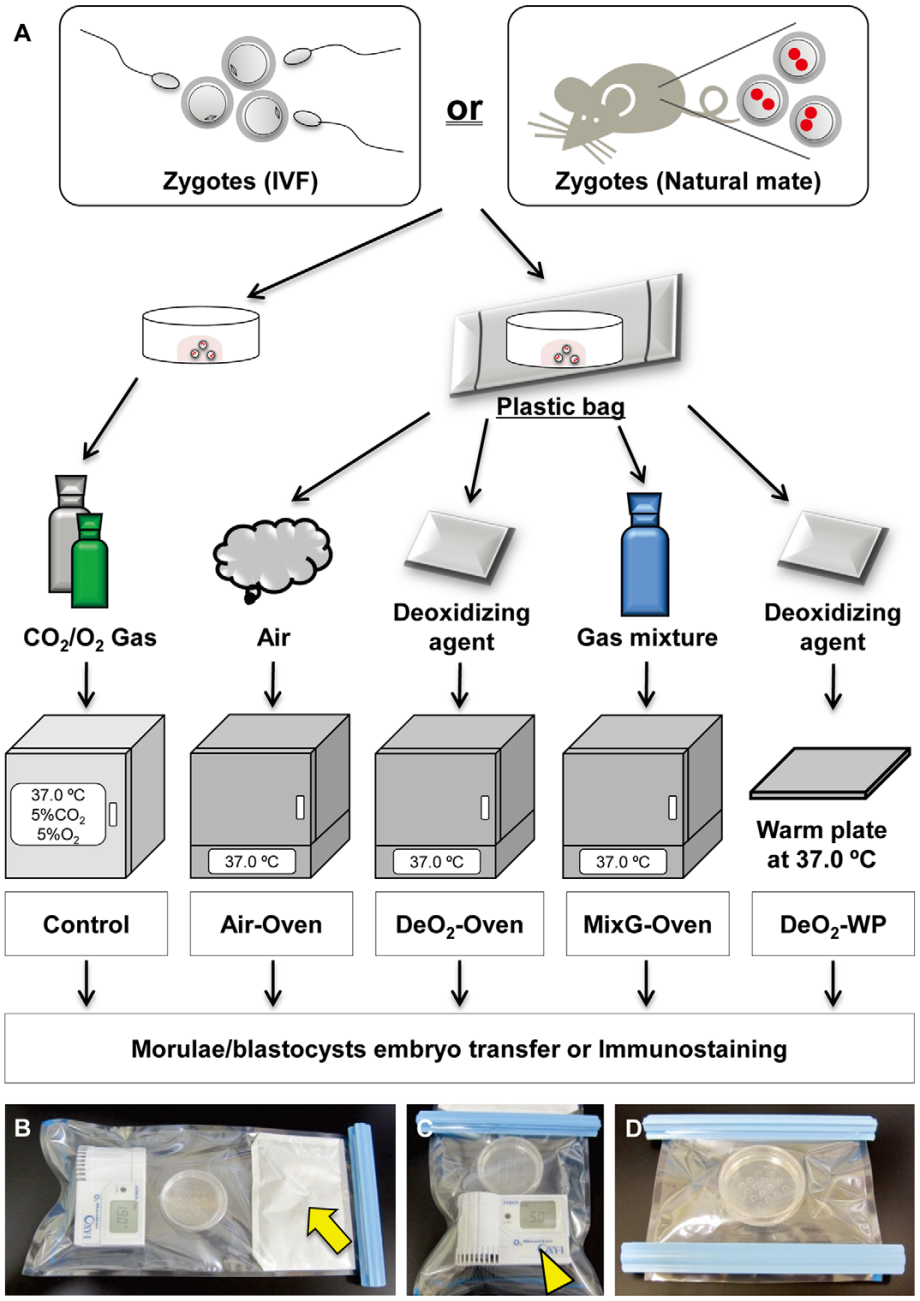
Experimental procedure and culture systems. (A) Schematic diagram of the experimental procedure and each culture system. Air-Oven experiment: the bag was filled with air and kept in an oven at 37°C. DeO_2_-Oven experiment: the deoxidizing agent was put into the bag, which was then kept in the oven. MixG-Oven experiment: the bag was filled with a gas mixture (see methodology) and kept in the oven. DeO_2_-WP experiment: the deoxidizing agent was put into the bag, which was then placed on a warming plate at 37°C. (B) The hermetically sealed plastic bag with a deoxidizing agent is indicated by an arrow. (C) The O_2_ concentration was regulated by the deoxidizing agent and measured using the oxygen meter (arrowhead). (D) After removal of the deoxidizing agent from the plastic bag.

The concentration of O_2_ is also very important for preimplantation embryo development [4], [5]. Methods for regulating this gas using chemical agents have been investigated in a hypoxic culture system [20], which was made simply in a plastic jar or bag using deoxidizing agents for absorbing O_2_. However, at the time of those investigations, it was difficult to monitor and regulate the concentration of O_2_ inside the system and the culture environment became excessively hypoxic, a condition that is known to inhibit embryo development [21]. Since then, miniaturized O_2_ meters have been produced that can be used in small chambers to monitor and regulate optimal O_2_ concentrations for embryo culture.

**Figure 2:**
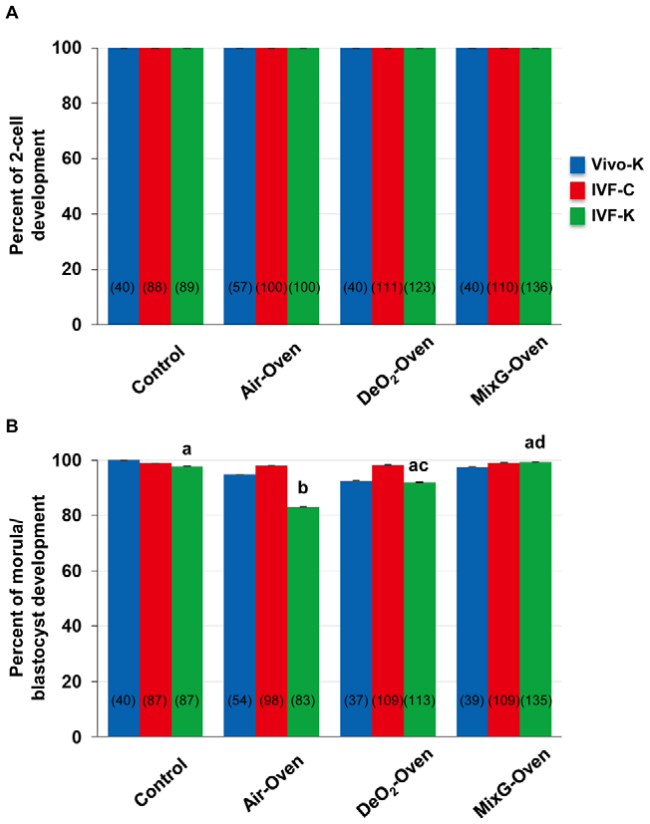
Preimplantation development rates of embryos derived from IVF and cultured under each culture system. Rates of development to the 2-cell (A) and morula/blastocyst (B) stages for each culture system. Vivo-K: Zygotes produced *in vivo* and cultured in KSOM. IVF-C: IVF-derived zygotes cultured in CZB medium. IVF-K: IVF-derived zygotes cultured in KSOM. Within columns, values with different letters are significantly different (*P*<0.05, χ^2^ tests).

Although there are several reports on performing embryo culture without an incubator or by adjusting the gas phase using chemical reagents, most of those experiments were performed to analyze blastocyst development, quality and/or gene expression and were unable to judge embryo quality accurately. The strongest evidence of good-quality embryos is the production of live offspring. With this objective, we examined preimplantation development in mouse zygotes under 5% O_2_ regulated by using a deoxidizing agent in a plastic bag and kept at 37°C without using a traditional incubator. To evaluate the embryos, some were examined for blastocyst quality in terms of cell numbers, gene expression and apoptosis rate by immunostaining and terminal deoxynucleotidyl transferase dUTP nick end labeling (TUNEL) assays; others were transferred into pseudopregnant recipient females to test their potential for development to full term. Randomly selected offspring were allowed to grow to adulthood and were mated to test their fertility.

**Figure 3:**
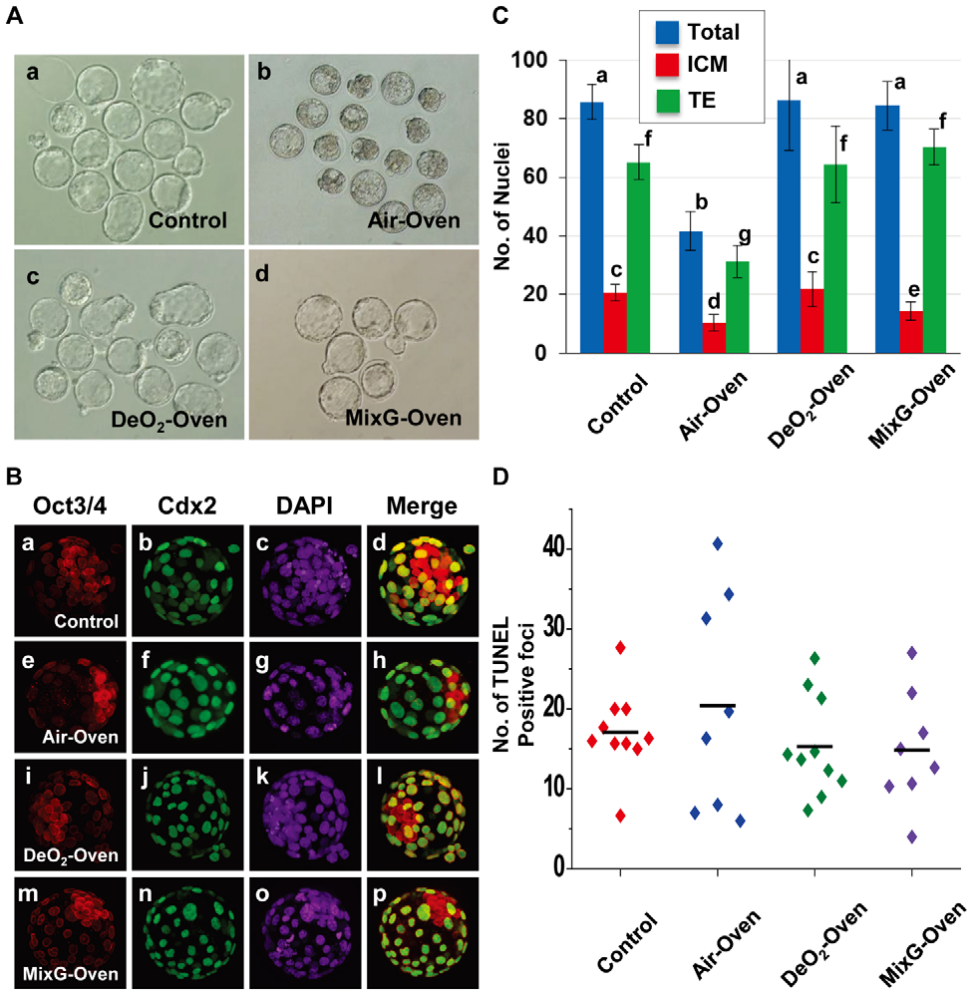
Blastocyst quality after culture in each culture system. (A) Expanded blastocysts cultured for 96 h under each culture system. (a) Control, (b) Air-Oven, (c) DeO_2_-Oven and (d) MixG-Oven. (B) Immunostaining of blastocysts at 96 h after IVF for Oct3/4 (red) (a, e, i, m), a marker for the inner cell mass (ICM); for CDX2 (green) (b, f, j, n), a marker for trophectoderm (TE); and with DAPI for staining DNA (c, g, k, o). Merged images are shown for Oct3/4 and CDX2 (d, h, i, p). (a–d) Control. (e–h) Air-Oven: the bag was filled with air and kept in the oven at 37°C. (i–l) DeO_2_-Oven: the deoxidizing agent was put into the bag, which was then kept in the oven. (m–p) MixG-Oven: the bag was filled with a gas mixture (see methodology) and kept in the oven. Original magnification ×400. (C) Numbers of ICM and TE cells compared between culture systems. (D) Numbers of apoptotic cells in blastocysts cultured in each culture system. Within columns, values with different letters are significantly different (*P*<0.05, Student's *t*-tests).

## Materials and Methods

### Animals

Female and male B6D2F1 (C57BL/6J × DBA/2) mice and C57BL/6N mice (2–3 months old) were obtained from Shizuoka Laboratory Animal Center (Hamamatsu, Japan). All animal experiments were conducted according to the Guide for the Care and Use of Laboratory Animals and were approved by the Institutional Committee of Laboratory Animal Experimentation of the RIKEN Kobe Institute (approval no. AH14–13–19).

**Table 1:** Full-term development of embryos fertilized *in vitro* and cultured in a plastic bag without using an incubator.

Treatment	Conc. of CO_2_	Conc. of O_2_	Culture medium	No. of cultured embryos	No. (%) of morulae/ blastocysts	No. of embryos transferred	No. (%) of embryos implanted	No. (%) of offspring	Mean body weight (g)	Mean placental weight (g)	Fertile*
Control	5%	5%	CZB	88	87 (99)	71	43 (60)^a^	31 (44)^a^	1.27±0.17	0.12±0.03	Yes
			KSOM	89	87 (98)	70	41 (59)	30 (45)	1.11±0.21^a^	0.12±0.02	Yes
Air-Oven	0.03%	20%	CZB	100	98 (98)	80	28 (35)^b^	21 (26)^b^	1.29±0.13	0.11±0.01^a^	Yes
			KSOM	100	83 (83)	80	38 (48)	25 (31)^c^	1.27±0.15^b^	0.11±0.03	Yes
DeO_2_-Oven	Not controlled	5%	CZB	111	109 (98)	109	76 (70)^a^	45 (41)^a^	1.29±0.25	0.11 ± 0.03^a^	Yes
			KSOM	123	113 (92)	99	59 (60)	48 (49)^d^	1.27±0.16^b^	0.12±0.02	Yes
MixG-Oven	5%	5%	CZB	110	109 (99)	100	46 (46)	37 (37)	1.36±0.16	0.13±0.03^b^	Yes
			KSOM	136	135 (99)	80	45 (56)	30 (38)	1.30±0.14^b^	0.13±0.02	Yes

* For fertility testing, three pairs were selected from each group at random.

a vs. ^b^, ^c^ vs. ^d^; values with different superscript letters are significantly different (*P*<0.05 by χ^2^ tests or Student's *t*-tests).

### Production of Zygotes from *In Vitro* Fertilization (IVF) or Natural Mating

Spermatozoa were collected from the cauda epididymidis of B6D2F1 males (>12 weeks) into 200 µl drops of human tubal fluid [22] medium covered with sterile mineral oil and capacitated by incubation for 1–2 h at 37°C under 5% CO_2_ in air. During sperm preincubation, cumulus–oocyte complexes (COCs) were collected from the oviducts of B6D2F1 female mice (8–12 weeks old) that were induced to superovulate by consecutive injections of equine chorionic gonadotropin (5 IU) and human chorionic gonadotropin (5 IU) 48 h apart. Sixteen hours after the human chorionic gonadotropin injection, the mice were killed to collect COCs. After sperm preincubation, 5 µl aliquots of the suspension were added to droplets of human tubal fluid containing COCs. The final sperm concentration of this method was about 2×10^5^ cells/ml. At 1.5 h after IVF, cumulus cells were dispersed by brief treatment with hyaluronidase (Type-IS, 150 units/ml, Sigma-Aldrich, St Louis, MO, USA). Oocytes were collected from the droplets and washed in Chatot–Ziomek–Bavister (CZB) [23] or KSOM [24] media (Chemicon Specialty Media, Phillipsburg, NJ, USA). *In vivo*-generated 1-cell zygotes were collected from the oviducts of superovulated B6D2F1 females following natural mating with fertile male mice. The zygotes were placed in fresh droplets of CZB or KSOM preincubated at 37°C under 5% CO_2_ in air and cultured for subsequent experiments.

**Figure 4:**
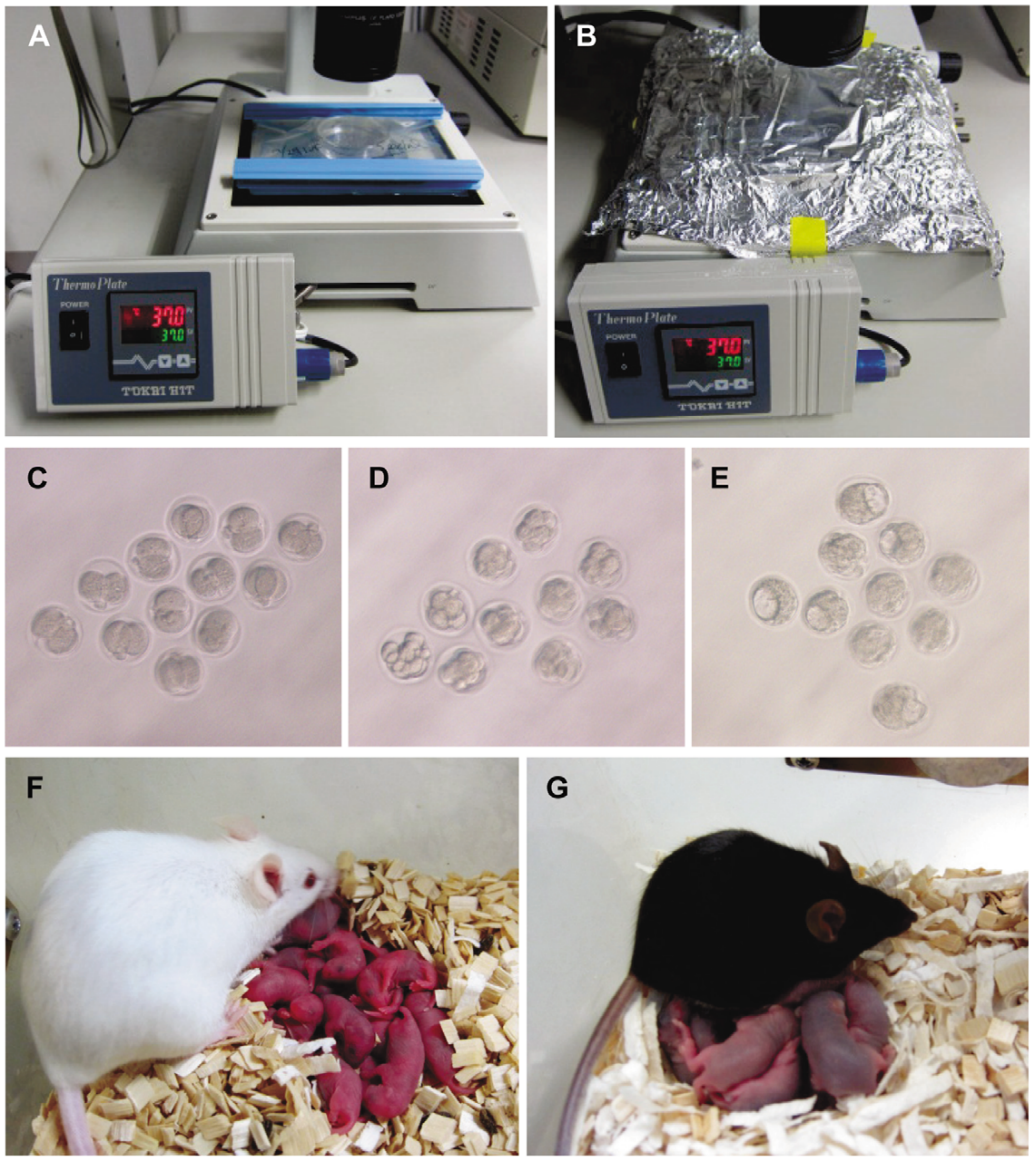
DeO_2_-WP culture system and development of full-term offspring. (A, B) The culture dish was placed in a sealed plastic bag with a controlled O_2_ concentration, placed on a warm plate at 37°C and then covered with aluminum foil during the culture period. (C) Two-cell-stage embryos cultured for 24 h; (D) 8-cell-stage embryos cultured for 48 h and (E) morulae/blastocysts cultured for 72 h on the warm plate. Because the plastic bag was transparent, it was possible to observe embryos directly using an inverted microscope. (F) Mice derived from embryos cultured in the DeO_2_-WP culture system. (G) After two or three months, these offspring had grown to adulthood and randomly selected mice were proven fertile by natural mating.

**Table 2:** Full-term development of embryos cultured in a plastic bag using a warming plate.

Treatment	Controlled gas conc.	Conc. of CO_2_	Conc. of O_2_	Humidity	No. of cultured embryos	No. (%) of 2-cell embryos	No. (%) of morulae/ blastocysts	No. of embryos transferred (recipients)	No. (%) of offspring
Control	Mixture*	5%	5%	100%	30	30 (100)	30 (100)	30 (2)	13 (43)
DeO_2_-WP	Deoxidizing agent	Not controlled	5%	Not controlled	44	44 (100)	44 (100)	44 (2)	20 (46)

* 5% CO_2_, 5% O_2_ and 90% N_2._

### Embryo Culture System

The zygotes were transferred to 30 µl drops of culture medium covered with sterile mineral oil on a culture dish and placed in a plastic bag (Mitsubishi Gas Chemical Co., Tokyo, Japan). Then, a capsule of deoxidizing agent (Sugiyama-Gen Co., Tokyo, Japan) was opened and placed in the plastic bag together with an oxygen meter (OXY-1, JIKCO CO., Tokyo, Japan). The bag was sealed immediately with a clip (Fig. 1B). Because the deoxidizing agent used for O_2_ absorption and the CO_2_ gas-forming agent both start reacting on contact with air, it was necessary to open these kits just before they were used. Using the oxygen meter for monitoring, a low O_2_ concentration (approximately 5%) was recorded in the plastic bag after about 5 min. According to the manufacturer, the plastic bag with the deoxidizing agent is normally left to stand for approximately 15 min to achieve a decrease to 5% O_2_. To stop further absorption of O2, a second clip was applied to the bag between the deoxidizing agent and culture dish to separate them (Fig. 1C), and then the deoxidizing agent and oxygen meter were removed using a third clip (Fig. 1D). Although this deoxidizing agent cannot regulate CO_2_ concentration, according to the manufacturer, the concentration of CO_2_ in the plastic bag should have been around 3%. The hermetically sealed plastic bags were kept at 37°C in air (we called this the DeO_2_-Oven experiment as we used a common hybridization oven in our laboratory). To determine the effect of the deoxidizing agent, the air in the plastic bag was aspirated and replaced with a gas mixture (5% CO_2_, 5% O_2_ and 90% N_2_) using a syringe. This was repeated twice and then the bag was placed inside the oven (MixG-Oven experiment). As a negative control, embryos were cultured in plastic bags with air at 37°C (Air-Oven experiment). Humidity was not regulated in the plastic bags. A positive control experiment was run using dishes cultured at 37°C under 5% CO_2_, 5% O_2_ and 90% N_2_ in 100% humidity in a traditional incubator without using a plastic bag (Control experiment). In addition, some embryos of the B6D2F1 strain were cultured on a warming plate at 37°C after being sealed in a plastic bag following the deoxidizing treatment and covered with aluminum foil (DeO_2_-WP experiment). To determine the effect of this simple incubator-free system on embryos from an inbred strain, some embryos from C57BL/6N mice were cultured using similar methods. The rates of development to 2-cell embryos or to the morula or blastocyst stage at 72 h were calculated from all the zygotes collected.

**Table 3:** Preimplantation development of inbred strain (C57BL/6) mouse embryos cultured in a plastic bag without using an incubator.

Treatment	Controlled gas conc.	Conc. of CO_2_	Conc. of O_2_	Humidity	No. of cultured embryos	No. (%) of 2- cell embryos	No. (%) of morulae/ blastocysts
Control	Mixture*	5%	5%	100%	30	30 (100)	30 (100)
DeO_2_-WP	Air with deoxidizing agent	Not controlled	5%	Not controlled	100	99 (99)	93 (93)

* 5% CO_2_, 5% O_2_ and 90% N_2._

### Embryo Transfer

Morula/blastocyst-stage embryos derived from each culture system were transferred into the uterus of pseudopregnant ICR strain female mice at 2.5 days post copulation. These had been mated with vasectomized ICR males. Six to 10 embryos were transferred into each uterus [25]–[27]. Offspring were obtained at 18.5 days post copulation through Cesarean section or by natural birth, and we recorded the body and placental weights and sex. Selected offspring were fostered to other ICR female mice to allow them to grow to adulthood. When they matured sexually, randomly selected males and females (three each) were paired and mated to test their fertility.

### Immunostaining and TUNEL Assay

Blastocyst-stage embryos cultured for 96 h after IVF in each culture system were fixed immediately after opening each plastic bag. The numbers and localizations of the inner cell mass (ICM) and trophectoderm (TE) cells were estimated by immunostaining. Briefly, blastocysts were fixed in phosphate-buffered saline (PBS) containing 4% paraformaldehyde for 30 min at room temperature. The fixed blastocysts were washed twice in PBS containing 1% (w/v) bovine serum albumin (BSA) and transferred into 1% BSA–PBS containing 0.1% Triton X-100 (Nacalai Tesque, Kyoto, Japan) and incubated overnight at 4°C. After blocking, the blastocysts were further incubated with the primary antibody diluted in blocking solution at 4°C for overnight. The primary antibodies used were rabbit polyclonal anti-Oct3/4 (POU5F1; 1∶100 dilution; Santa Cruz Biotechnology, Inc., Tokyo, Japan) for detecting ICM cells and anti-CDX2 mouse monoclonal antibody (1∶100 dilution; BioGenex, Inc., San Ramon, CA, USA) for detecting TE cells. After the embryos had been washed twice in the blocking solution, they were incubated for 1 h with fluorescent dye-conjugated secondary antibodies: Alexa Fluor 564-labeled goat anti-rabbit IgG and Alexa Fluor 488-labeled goat anti-mouse IgG (1∶200 dilution; Molecular Probes, Inc., Eugene, OR, USA). After the embryos had been washed twice in 1% BSA–PBS, the DNA was stained with 4′,6-diamidino-2-phenylindole (DAPI) (2 µg/ml; Molecular Probes, Inc).

For the TUNEL assay of apoptosis, blastocysts collected at 96 h after IVF were fixed and washed twice in 1% BSA–PBS. TUNEL analysis was performed using the Fluorescein In Situ Cell Death Detection Kit (Roche, Boehringer-Mannheim, Germany) following the manufacturer's recommendations. After the TUNEL reaction, blastocysts were washed three times, blocked and immunostained for CDX2 to detect TE cells and stained with DAPI to detect nuclear DNA.

The embryos were transferred to 2 µl drops of PBS containing 0.5% (w/v) polyvinylpyrrolidone (Sigma-Aldrich) in a glass-bottomed dish, observed under an inverted fluorescence microscope (IX-71, Olympus, Tokyo, Japan) equipped with a Nipkow disk scanning confocal unit (CSU X-1, Yokogawa Electric Corp., Japan) [25], [26] and exposed to three different wavelengths of excitation (405, 488 and 561 nm). Images sectioned optically at 1 μm intervals were acquired in the Z-axis and three separate color images (blue, green and red) were captured. Three-dimensional images of the embryos were reconstructed using MetaMorph software (Molecular Devices, Sunnyvale, CA, USA).

### Time-Lapse Live Embryo Imaging

Time-lapse live embryo imaging was performed using our previous method with slight modifications [25], [26]. Briefly, pronuclear-stage oocytes were transferred to 5 µl drops of KSOM medium on a glass-bottomed dish and sealed in a plastic bag after deoxidizing treatment. The plastic bag was set on the microscope stage with a warming plate and time-lapse light images were captured at 15 min intervals until the blastocyst stage.

### Statistical Analysis

Outcomes were evaluated using χ^2^ tests or Student's *t*-tests and *P*<0.05 was regarded as statistically significant.

## Results

### Regulation of O_2_ Concentration by the Deoxidizing Agent

In the standard method recommended by the manufacturer, the plastic bag is left to stand for approximately 15 min to reach 5% O_2_. However, in this study, we tried to enhance the action of the deoxidizing agent by gently massaging the plastic bag. When the deoxidizing agent was sealed in the plastic bag (Fig. 1B–D), the concentration of O_2_ decreased immediately and reached 5% approximately 5 min later. Although this method was easy, the O_2_ concentration varied a little (approximately 4.5–5.5%). However, this concentration range had no effect on embryo development, consistent with the findings of a previous report [28]. Moreover, in a preliminary experiment, we found that the plastic bag and clips did not absorb any external O_2_ for at least 5 days as monitored using the O_2_ meter (data not shown).

### Effect of Each Culture System on Development In Vitro

In this study, we used two culture media (CZB and KSOM) and two kinds of embryos (derived *in vivo* and by IVF) to evaluate the flexibility of our method. As shown in Fig. 2A, after culturing the IVF-derived or *in vivo*-fertilized zygotes for 24 h, all of the embryos reached the 2-cell stage in all experimental conditions and the development rates did not differ significantly from the controls. When embryos were cultured for 72 h, although the morula/blastocyst formation rate in the Air-Oven experiment cultured in KSOM was slightly lower than other groups (*P*<0.05; Fig. 2B), most embryos reached the morula/blastocyst stage (83.0–99.3%, Fig. 2) with similar rates to conventional culture systems [26], [29]–[31]. These results suggest that mouse embryos can be cultured *in vitro* using a plastic bag without the need for a traditional incubator.

### Comparison of the Quality of Blastocysts between Culture Systems

It is well known that the rate of blastocyst development alone is not enough to demonstrate the normality of embryos because even genetically damaged embryos can develop to this stage [32]. As shown in Fig. 3, the quality of blastocysts was evaluated based on the cell number, cell differentiation and allocation of ICM cells, and on the incidence of apoptosis using immunostaining and TUNEL assays, respectively. In the Air-Oven system, the total ICM and TE cell numbers were significantly lower than those obtained using the other culture systems (*P*<0.05; Fig. 3B, C). However, in the DeO_2_-Oven system, the total numbers of both cell types were not decreased significantly compared with controls. In the MixG-Oven, although the ICM number was significantly lower than in control embryos (*P*<0.05), the total number of all blastomeres was similar to that in the control experiment. Interestingly, even with these differences, the polarity of the ICM in blastocysts and the incidence of apoptosis were not significantly different between any of the culture systems (Fig. 3D).

### Potential for Full-Term Development after Embryo Culture

In demonstrating the normality of embryos, the strongest evidence is to show the potential for full-term development. After culturing IVF-derived embryos, morulae or blastocysts were transferred into pseudopregnant ICR strain female mice. As shown in Table 1, embryos derived from either the DeO_2_-Oven or MixG-Oven culture systems developed to full term, with a success rate similar to control embryos (37–48% vs. 43%). The different culture media used had no effect on the success rates of producing offspring. Interestingly, even embryos from the Air-Oven system developed to full term. Although the success rate in this group was significantly lower than in the others, it suggests that some embryos are very resilient to poor environments (see Fig. 3D; Air-Oven). We also examined the potential for full-term development of *in vivo*-fertilized embryos. The full-term development rates in the DeO_2_-Oven, MixG-Oven and Air-Oven systems were all similar to controls at 20/40 (50%), 20/40 (50%) and 19/40 (48%), versus 18/40 (45%), respectively. Randomly selected offspring from all experimental groups grew to adulthood and these mice proved fertile when mated with each other.

### Embryo Culture on a Warm Plate

To simplify the culture system, we used a warm plate to maintain the culture temperature, instead of an oven (Fig. 4A, B). As shown in Table 2, even when cultured in this simple manner, all the embryos developed to the morula/blastocyst stages (Fig. 4C–E) and 20 healthy offspring (46%) were obtained after transfer into recipient females (Fig. 4F). This success rate was similar to the control group (43%). All these mice grew to adulthood and were fertile (Fig. 4G).

To demonstrate the utility of our simple culture system, we also examined inbred strain (C57BL/6N) embryos instead of B6D2F1 embryos. With these, 99% of the embryos reached the 2-cell stage and 93% of embryos reached the morula/blastocyst stage at 72 h after *in vitro* culture, similar to the controls (Table 3).

### Time-Lapse Live Embryo Imaging

As it was possible to observe the developing embryos through the transparent wall of the bag, we sought to use this method as an easy live imaging system when combined with a time-lapse microscope without using an incubation chamber. When B6D2F1 zygotes were cultured by this system, most embryos developed to blastocysts, as observed by time-lapse microscope for 4 days. As shown in Video S1, pronuclei of zygotes, blastomeres of embryos and cell division were clearly observed in all embryos.

## Discussion

We obtained healthy offspring successfully from embryos cultured without a traditional incubator, simply by using a plastic bag kept at 37°C. The success rate was increased significantly to almost the same rate as that obtained using traditional culture methods when a deoxidizing agent was used to adjust the O_2_ concentration.

Several attempts to simplify the culture method of mammalian embryos have been reported, such as culture in a water bath [9], culture in air or culture without humidity control [12]. However, many reports showed poor embryo development in such conditions. Arias et al. reported that bovine blastocysts cultured in foil and plastic bags filled with a gas mixture and kept in a 5% CO_2_, 5% O_2_ and 90% N_2_ incubator showed significantly reduced cell numbers and increased apoptosis compared with those cultured in an incubator chamber [11]. Swain reported that when cultured under air alone (approximately 0.03% CO_2_), the pH of the medium was 8.1–8.3 and the embryos did not reach the blastocyst stage [12]. Ozawa et al. also reported that a high pH in the medium caused a lower development rate and poorer embryo quality [13]. Therefore, it has been thought that mammalian embryos are extremely sensitive to variations in culture conditions and that careful adjustment of the gas phase and use of a CO_2_ incubator are essential to obtain good-quality embryos after prolonged culture.

In this study, when mouse embryos were cultured in a plastic bag with air, the rate of development to the blastocyst was reduced, as in previous reports [12]. Surprisingly, after transferring these embryos to recipient female mice, we obtained offspring with a relatively high success rate. It is not clear why such poor-quality embryos could develop to full term and why we succeeded and others failed. Probably it is because we used commercially available Mitsubishi Gas Chemical plastic bags, which had already been demonstrated by the company to have no cellular toxicity. If embryos are very sensitive to chemicals leaching from plastics, these might inhibit embryo development. In addition, because the bags we used are transparent, it was possible to observe the embryos through the wall without the culture dish being removed (Fig. 4C–E). Thus, the bag protected the dish and embryos from frequent changes in temperature and gas composition during observation. Once embryos develop to blastocysts, even if their quality is poor at the time of transfer into pseudopregnant recipients, they can recover while in the uterus before implantation [33]. Thus, although it has long been believed that mammalian embryos are sensitive to the culture environment, if we can protect against factors such as frequent changes in temperature or gas composition, even long-term embryo culture is no longer difficult.

The success rate of producing live offspring was very high, even when embryos were cultured in a plastic bag with the concentration of O_2_ reduced to 5%. The success rate was almost the same as with a traditional culture method, and the offspring look normal and are fertile. Interestingly, when we compared the use of a deoxidizing agent with a gas mixture, the blastocyst formation and offspring production rates were slightly higher in the former method. The deoxidizing agent absorbs O_2_ and releases CO_2_, but only the concentration of O_2_ could be monitored and controlled using the oxygen meter. According to the manufacturer, when the deoxidizing agent reduces the concentration of O_2_ to 1%, the concentration of CO_2_ will be 5%. Therefore, the concentration of CO_2_ in this study was probably lower than 5%. On the other hand, the gas mixture in a traditional incubator always contains 5% O_2_ and 5% CO_2_. This suggests that 5% CO_2_ is an effective concentration for supporting normal development. However, if embryos are cultured under 5% O_2_, the appropriate concentration of CO_2_ might be less than 5%.

From an economical point of view, using a deoxidizing agent to adjust the O_2_ concentration is easier and cheaper than using a commercial gas mixture. Moreover, as shown in Tables 2 and 3, embryos from several mouse strains were cultured successfully simply on a warm plate at 37°C using this culture system, so this could be applied for moving embryos between locations. Recently, several methods for embryo transportation have been reported. Human oocytes and embryos have been transported between two IVF centers using portable but heavy CO_2_ incubators with no detrimental effects on fertilization or pregnancy rates [34], [35]. Chen et al. also reported that mouse oocytes could be transported to another facility using an incubator without a CO_2_ supply, but the incubation time was only 5 h [36]. Our results suggest that if we maintain the temperature at 37°C for 3–4 days using a simple heating system, embryos could grow and be transported without their quality being compromised. In addition, with this system, it is possible to observe the developing embryos through the transparent wall of the bag. This feature allowed us to observe live embryos by time-lapse imaging without using an expensive CO_2_ incubation chamber on the microscope. Thus, this system will be very useful not only as a simple and cheap embryo culture approach, but also as a cost-effective method for international transportation of embryos without the need for special care.

## Supporting Information

Video S1 **This shows embryo development observed using time-lapse microscopy from the pronuclear to blastocyst stages without using a specialized incubation chamber.**
(AVI)
